# Effect of Probiotic *Bifidobacterium bifidum* TMC3115 Supplementation on Psychosocial Stress Using a Sub-Chronic and Mild Social Defeat Stress in Mice

**DOI:** 10.3390/nu14050970

**Published:** 2022-02-24

**Authors:** Kazutoyo Yoda, Gaku Harata, Mizuho Sato, Kenji Miyazawa, Natsuki Ohsawa, Fang He, Atsushi Toyoda

**Affiliations:** 1 Technical Research Laboratory, Takanashi Milk Products Co., Ltd., 5 Honjuku-cho, Asahi-ku, Yokohama 241-0023, Japan; g-harata@takanashi-milk.co.jp (G.H.); ke-miyazawa@takanashi-milk.co.jp (K.M.); ka-hou@takanashi-milk.co.jp (F.H.); 2 College of Agriculture, Ibaraki University, 3-21-1 Chuo, Ami, Inashiki 300-0393, Japan; mizuho.sato.0519@gmail.com (M.S.); obutvlj.729@gmail.com (N.O.); atsushi.toyoda.0516@vc.ibaraki.ac.jp (A.T.); 3 United Graduate School of Agricultural Science, Tokyo University of Agriculture and Technology, 3-8-1 Harumi-cho, Fuchu, Tokyo 183-8509, Japan

**Keywords:** *Bifidobacterium*, probiotics, stress, interleukin-6

## Abstract

With the accumulation of knowledge on the relation between psychological stress and gut microbiota, there is growing interest in controlling stress and/or mood disorders via probiotic supplementation. We aimed to examine the effect of probiotic *Bifidobacterium bifidum* TMC3115 (TMC3115) supplementation using a sub-chronic and mild social defeat stress murine model in this study. TM3115 supplementation maintained body weight gain and alleviated a polydipsia-like symptom induced by the stress. In the analyses of fecal and cecal bacterial profiles, expansions of *Proteobacteria* in stressed mice and increases in *Actinobacteria* and *Bifidobacterium* in mice supplemented with TMC3115 were observed. There was no marked difference in the diversity of cecal bacteria between the tested mice. Elevated serum levels of inflammatory markers such as tumor necrosis factor (TNF)-α and interleukin (IL)-6 were observed in the stressed mice, while TMC3115 only reduced the IL-6 level. These findings suggest that TMC3115 supplementation confers tolerance to psychosocial stress in the host through modulation of the gut microbiota and alleviation of stress-induced inflammatory responses. Furthermore, it may be expected to exert prevention and treatment of disorders related to peripheral IL-6, including depression.

## 1. Introduction

Stressful life events often lead to a variety of disorders, such as depression, post-traumatic stress disorder, and cardiovascular disease [1]. Stress induces psychological and physiological changes, including activation of the hypothalamic–pituitary–adrenal axis and sympathetic nervous system [2], which can significantly affect mood, behavior, and health. The recent growing research interest in the brain–gut axis has also revealed that stress alters the gut microbiota [3,4]. In other words, not only alleviating stressful conditions and protecting ourselves from stress, but also maintaining good intestinal conditions, could be effective approaches to reducing the risk of developing these disorders.

As one of the definitions of probiotics is “A live microbial feed supplement which beneficially affects the host animal by improving its intestinal microbial balance” [5], the current strategy of probiotics restores microbial diversity and alters the perturbed intestinal microbiota [6]. So far, many pieces of research have shown that probiotics have a wide range of health benefits [7], for instance, the alleviation and/or prevention of allergies [8,9] and infectious diseases [10,11,12]. In addition, from the viewpoint of the brain–gut axis, there are growing expectations for the prevention and treatment of psychiatric disorders, such as depression, anxiety, and schizophrenia [13].

The chronic social defeat stress (CSDS) model is often used for studying depression because mice subjected to CSDS display various depressive-like features [14]. Several studies have tried to examine the effects of probiotic administration on stress and depression using the CSDS model [15,16]. Goto et al. [17] developed a sub-chronic and mild CSDS (sCSDS) model to reduce physical stress from the standard CSDS protocol (5 to 10 min of physical contact with aggressors per day) [18]. It has been reported that microbiota in the cecum and feces of mice subjected to the sCSDS model is different from that of non-stressed control mice [19]. Therefore, some studies assessed the effects of probiotic or postbiotic [20] lactobacilli, but not bifidobacteria, on stress-induced behavioral, molecular, and/or intestinal microbiota changes using the sCSDS model [21,22,23].

*Bifidobacterium bifidum* TMC3115 (TMC3115) is a probiotic bacterium originally isolated from healthy infant stools [24]. It is thought that TMC3115 exerts anti-obesity and anti-allergic effects by acting on the intestinal microbiota and intestinal immune system [25,26,27]. Although TMC3115 can directly affect hippocampal neurons in vitro [28], there are no findings of the effects of TMC3115 administered orally on stress, behavior, and brain–gut axis. In the present study, we aimed to investigate the effects of oral supplementation of TMC3115 on mice subjected to sCSDS.

## 2. Materials and Methods

### 2.1. Bacterial Strain

*Bifidobacterium bifidum* TMC3115 (TMC3115) was provided from the Takanashi Microorganisms Collection (Takanashi Milk Products Co., Ltd., Kanagawa, Japan). Food grade (FG) medium described previously by Miyazawa et al. [29] was used to culture TMC3115 (Table S1). The cultured bacterial cells were collected by centrifugation (8000 g, 8 min, 4 °C) and washed twice with sterile water. After washing, the collected bacteria were lyophilized and then kept at −80 °C until use. The viable bacterial cell count in the lyophilized powder was more than 10^10^ cells/g.

### 2.2. Animals

Male C57BL/6JJcl (B6) mice aged 7 weeks were purchased from CLEA Japan, Inc. (Tokyo, Japan). Male ICR mice aged more than 6 months (retired from breeders) were obtained from Japan SLC, Inc. (Shizuoka, Japan). The mice were individually housed in cages with wood-shaving bedding under controlled temperatures (22 ± 1 °C) and a 12 h light–dark cycle (light phase, 7:00 a.m.–7:00 p.m.), with access to food and tap water ad libitum. An AIN-93G diet (powder type; Oriental Yeast Co., Ltd., Tokyo, Japan; Table S2) was fed to B6 mice using the Roden CAFE (Oriental Yeast Co., Ltd.), and a standard laboratory diet (MF diet; pellet type; Oriental Yeast Co., Ltd.) was fed to ICR mice. For B6 mice, the body weight, food, and water intake of B6 were recorded, and feed was exchanged each day. All mice were housed under the above conditions for 1 week for acclimation.

### 2.3. Experimental Design

**Figure 1:**
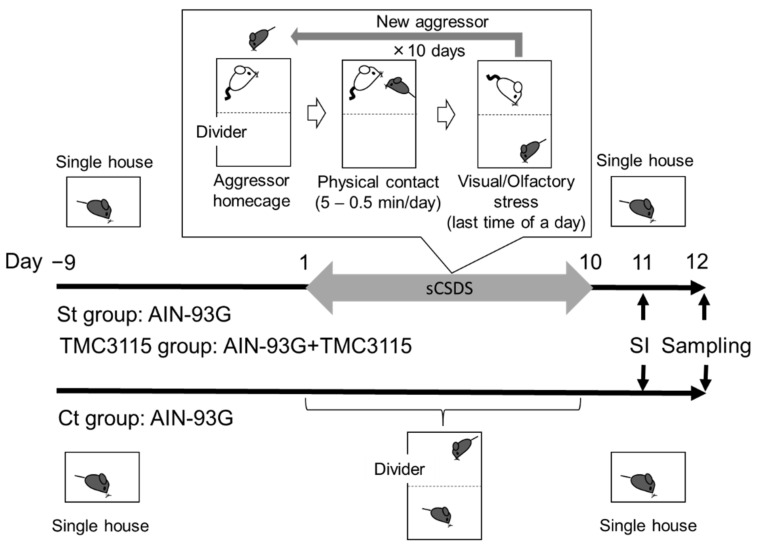
Experimental design. Mice in both the St and TMC3115 groups were subjected to the subchronic and mild social defeat stress (sCSDS) for 10 days. The behavior of all mice was observed by the social interaction (SI) test on Day 11.

After acclimation, the B6 mice were divided into three groups based on body weight: a control group fed AIN-93G without sCSDS (Ct group, *n* = 20), a control group fed AIN-93G and subjected to sCSDS (St group, *n* = 24), and an experimental group fed AIN-93G containing 0.42% TMC3115 lyophilized powder and subjected to sCSDS (TMC3115 group, *n* = 25). The mice were fed the respective diets throughout all test periods, including the 10 days of pre-treatment (Figure 1). All experiments were conducted between 24 July 2018 and 23 April 2019.

### 2.4. sCSDS Model

sCSDS was performed as previously described [30] (Figure 1). Briefly, the B6 mice (intruder) were exposed to a different ICR mouse (aggressor) each day for predetermined time for 10 days. The exposure time was set at 5 min after the first attack on Day 1 and then was reduced 0.5 min per day from Day 2 to Day 10. To check the aggressive behaviors in all sCSDS sessions, a commercially available video camera (Everio, JVC KENWOOD Corporation, Kanagawa, Japan) was used. After the sCSDS session, the resident ICR mouse and intruder mouse were housed in one half of the cage separated with a punched acrylic divider to allow visual, olfactory, and auditory contact for the remainder of the 24 h period. During the sCSDS period, Ct group mice were also housed in the same type of cage as the mice subjected to sCSDS; however, two Ct group mice were housed on the opposite sides of the acrylic divider.

### 2.5. Social Interaction Test

A social interaction (SI) test was performed on Day 11 (Figure 1). The protocol was basically conducted as described previously [30]. A wired-mesh plastic target box (100 mm × 100 mm × 130 mm) was placed in one side of an open-field arena (400 mm × 400 mm × 300 mm; OF-3002; O’Hara & Co., Tokyo, Japan), and the 6–7 cm wide area surrounding the target box was defined as an interaction zone. Two opposing corners of the interaction zone were defined as corner zones. A test C57BL/6J mouse was allowed to roam around the open-field arena for 2.5 min without an ICR mouse in the target box. After this, an unfamiliar ICR mouse was placed in the target box, and the test mouse was placed back into the open-field arena for 2.5 min. The total distance (cm) moved and the time spent in the interaction zone(s) and in the corner zone(s) were measured using Image SI software (O’Hara & Co.). SI scores were calculated as 100 × (interaction time, target present)/(interaction time, target absent) [31]. In the SI test, mice showing SI scores of less than 100 are generally defined as stress-susceptible, and those showing above it as stress-resilient.

### 2.6. Bacterial Analysis in Feces and Cecal Contents

To collect the feces, all mice were separately placed in clean empty cages for 30 min on Day 11. Feces were picked up with clean tweezers as soon as mice defecated, put into a sterilized tube and kept cold on ice during the collection, and then stored until use. To avoid unnecessary stress, the mice were immediately returned to each original house cage after 30 min regardless of whether they defecated or not. DNA extraction from feces and cecal contents was carried out using NucleoSpin^®^ DNA Stool kit (MACHEREY-NAGEL GmbH & Co., KG, Düren, Germany) based on the manufacturer’s instructions. The DNA concentration and quality of purified DNA were analyzed with the Qubit 3.0 fluorometer (Invitrogen, Waltham, MA, USA) and the TapeStation (Agilent Technologies Inc., Santa Clara, CA, USA). The 16S Library was constructed according to the ‘16S Metagenomic Sequencing Library Preparation protocol’ recommended by Illumina, Inc. (San Diego, CA, USA). PCR on a TaKaRa PCR Thermal Cycler Dice^®^ Touch (Takara Bio Inc., Shiga, Japan) was performed with 2 × KAPA HiFi HotStart ReadyMix (Kapa Biosystems, Inc., Wilmington, MA, USA) under the following conditions: initial denaturation at 95 °C for 3 min, followed by 25 cycles of 95 °C for 30 s, 55 °C for 30 s, and 72 °C for 30 s, and ended with an extension step at 72 °C for 5 min. The DNA concentration and size distribution of ready libraries were analyzed with the Qubit fluorometer and the TapeStation. The PCR products were purified using AMPure XP magnetic beads (Beckman Coulter, Inc., Brea, CA, USA), diluted into an equimolar concentration, and pooled according to their unique barcode sequence, which enables multiplexing. Next, Illumina dual-index barcodes were added to the pooled PCR products with the Nextera XT Index Kit (Illumina, Inc.). The indexed PCR products were purified and pooled into the equimolar concentration prior to paired-end sequencing with MiSeq Reagent Kit v3 (600-cycle) (Illumina, Inc.), following the manufacturer’s directions.

### 2.7. Sequencing Data Analysis

For the microbial sequence analysis, the low-quality sequences were filtered and chimeric sequences were removed by USEARCH (version 6.1.544). The QIIME (version 1.9.1) was used with default parameters for identifying representative sequences for each operational taxonomic unit (OTU) generated from complete linkage clustering with a 97% similarity and aligned to the Greengenes database (release 13_8). OTU tables with percent relative abundances were further processed at different taxonomic levels. Alpha-diversity calculations were performed and visualized with QIIME script core_diversity_analyses.py. Beta-diversity calculations were performed using QIIME 2 (version 2019.10.0.) and visualized using the principal coordinate analysis plots (PCoA), based on unweighted UniFrac, weighted UniFrac, with the QIIME 2 View (https://view.qiime2.org, accessed on 11 November 2021).

### 2.8. Measurement of Proinflammatory Cytokines in Serum

After the B6 mice were fasted for 3 h, blood samples were collected from the inferior vena cava under anesthesia with 3% isoflurane on Day 12. The whole blood was incubated at 37 °C for 20 min, kept at 4 °C for 2 h, and then centrifuged (1000 g, 4 °C, 15 min). The serum was collected and stored at −80 °C until use. The serum levels of the following inflammation markers were determined using appropriate enzyme-linked immunosorbent assay kits: TNF-α: #BMS607HS, IL-6: #BMS603HS, Invitrogen, following the manufacturer’s instructions.

### 2.9. Statistical Analysis

Statistical comparisons between three groups were performed using one-way or repeated two-way analysis of variance with Tukey’s post hoc test. A 2 × 3 cross table was analyzed with a chi-square test to detect differences in the resilience rates between groups. For sequencing data, statistical comparison was conducted by Kruskal–Wallis test with Bonferroni post hoc test. All statistical analyses were performed using R software (version 4.1.0, www.R-project.org, The R Foundation for Statistical Computing, Vienna, Austria). A difference with *p* < 0.05 was considered significant.

## 3. Results

### 3.1. Body Weight, Food Intake, and Water Intake

**Figure 2:**
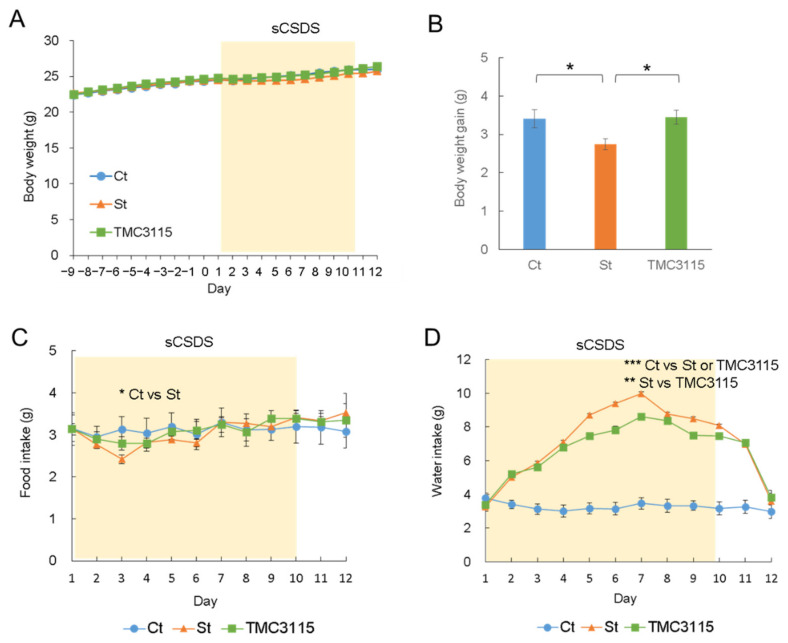
Body weight during the experiment (**A**), body weight gain after the experiment (**B**), and changes in food intake (**C**) and water intake (**D**) during the sCSDS period. Control mice (Ct group): circle; unsupplemented sCSDS mice (St group): triangle; sCSDS mice supplemented with TMC3115 (TMC3115 group): square. Repeated two-way ANOVA with Tukey’s post hoc test was conducted for (**C**,**D**). Bars represent mean ± SE. * indicates significant post hoc differences between groups, * *p* < 0.05, ** *p* < 0.01, *** *p* < 0.001.

The average body weight of each group during the experiment is shown in Figure 2A, and no significant difference between the groups was observed. On the other hand, the body weight gain (vs. the body weight at Day 9) after the sCSDS period significantly decreased in the St group compared with the Ct and TMC3115 groups (*p* = 0.045 and *p* = 0.021, respectively; Figure 2B). A significant lower food intake was observed in the St group compared with the Ct group during the sCSDS period (*p* = 0.027, Figure 2C). However, there was no significant difference in the total food intake through the experiment between the groups (Ct: 69.9 ± 1.5 g, St: 69.0 ± 1.0 g, TMC3115: 70.4 ± 1.3 g). Although the water intake of the TMC3115 group was lower than that of the St group during the sCSDS period (*p* = 0.003), both groups showed a greater water intake compared with the Ct group mice (*p* < 0.001; Figure 2D).

### 3.2. Social Interaction Test

The results of the social interaction (SI) test are shown in Table 1. Supplementation of TMC3115 reduced the ratio of stress-susceptible mice from 33.3% in the St group to 20% in the TMC3115 group, bringing it closer to the ratio in the non-stress control (15%). However, there were no significant differences in the number of stress-susceptible mice and the SI score among groups. The total distance (cm) in the St group was significantly shorter than that in the Ct group in the presence of ICR mice (*p* = 0.008), and the trend was observed in the absence of ICR mice (*p* = 0.066). There were no significant differences in interaction zone time and corner zone time among groups.

### 3.3. Bacterial Analyses in Feces and Cecal Contents

**Figure 3:**
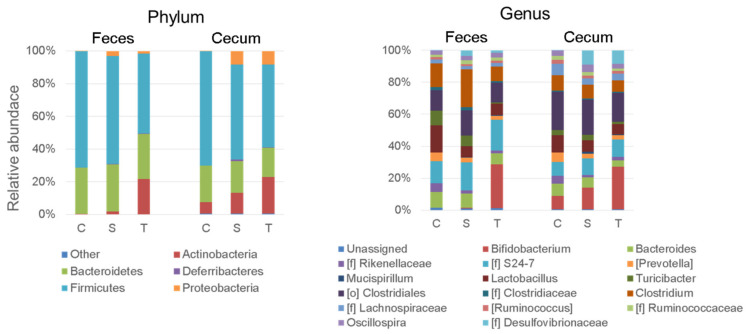
Bacterial composition in feces and cecal contents. C: control mice, S: unsupplemented sCSDS mice, T: sCSDS mice supplemented with TMC3115. The number of mice analyzing fecal bacteria on Day −9, Day 0, and Day 11 were C (*n* = 19, 18, and 18), S (*n* = 23, 23, and 19), and T (*n* = 19, 20, and 19), respectively. For cecal bacterial analysis, the number of mice were C (*n* = 20), S (*n* = 24), and T (*n* = 25), respectively.

Attempts were made to collect fecal samples from all individuals; however, this was not possible in some mice that did not defecate within the set 30 min. On the other hand, cecal contents were collected from all tested mice (a total of 69 mice). The compositions of bacterial groups with a median percent of 0.01 or higher in the feces and cecal contents are shown in Figure 3. At the phylum level, Bacteroidetes and Firmicutes were mostly dominant in all of the groups. Actinobacteria appeared except for the feces of the Ct group and was composed of the genus Bifidobacterium. The phyla Deferiibacteres and Proteobacteria were detected from only the mice subjected to sCSDS, but not the Ct group mice. The majority of them belonged to the genus Mucispirillum and the unclassified genus belonging to the family Desulfovibrionaceae ([f]_Desulfovibrionaceae), respectively. On the other hand, the abundances of each genera comprising Bacteroidetes and Firmicutes did not vary markedly regardless of the appearance of Actinobacteria and Proteobacteria.

**Figure 4:**
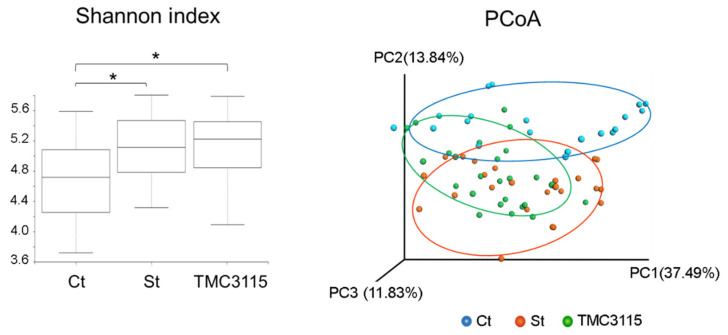
The α- and β-diversities of cecal microbiota. Ct: control mice, St: unsupplemented sCSDS mice, TMC3115: sCSDS mice supplemented with TMC3115. * indicates significant differences between groups, * *p* < 0.05.

In the analysis of the α-diversity of cecal microbiota, significant differences were found between the non-stressed group and the stressed groups (*p* < 0.05; Figure 4). The Ct and St group mice were divided into different clusters by the principal component analysis (PCoA) of β-diversity of cecal microbiota (Figure 4). The plots from the TMC3115 group mice were distributed across both clusters.

### 3.4. Tumor Necrosis Factor-α and Interleukin-6 Levels in Blood Serum

**Figure 5:**
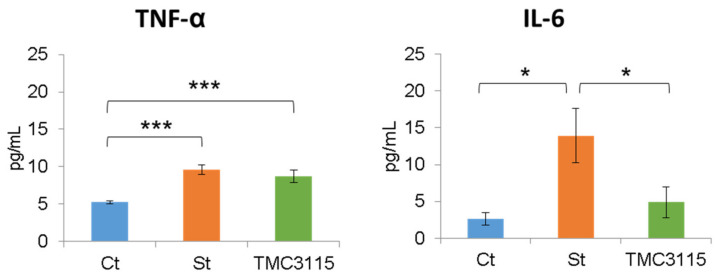
Serum levels of TNF-α and IL-6. Ct: control mice; St: unsupplemented sCSDS mice; TMC3115: sCSDS mice supplemented with TMC3115. Bars represent mean ± SE. * indicates significant differences between groups, * *p* < 0.05, *** *p* < 0.001.

The proinflammatory cytokine levels in the blood serum are shown in Figure 5. The tumor necrosis factor (TNF)-α levels of the St and TMC3115 groups were notably higher than that of the Ct group (*p* < 0.001, each). The interleukin (IL)-6 level of the St group remarkably increased compared with those of the Ct (*p* = 0.011) or TMC3115 groups (*p* = 0.036).

## 4. Discussion

In the present study, we examined the effect of TMC3115 supplementation on psychosocial stress using an sCSDS murine model and demonstrated to influence some aspects of stress-induced symptoms. Firstly, dietary TMC3115 intake recovered the lower body weight gain induced by sCSDS (Figure 2B). Stress affects body weight and weight change through several mechanisms, for instance, appetite and physical activity [32]. The decrease in food intake observed in the unsupplemented mice during the sCSDS period might generate a lower body weight gain (Figure 2C). In contrast, the mice supplemented with TMC3115 did not show such a decrease in food intake despite the stress load. Therefore, they might be able to maintain their appetite and body weight gain. Secondly, the TMC3115 supplementation alleviated the polydipsia-like symptom (excessive water intake) during the sCSDS period (Figure 2D). A polydipsia-like symptom is the most characteristic phenotype of the sCSDS model [17,21,22,23]. Although this phenotype is caused by stress, the mechanism behind this is still unknown. In humans, primary polydipsia is common in patients with neurodevelopmental disorders and chronic psychotic disorders [33]. However, it is also known that psychological stress becomes a trigger for primary polydipsia even in adolescents without psychiatric comorbidity [34]. Thus, TMC3115 supplementation is likely to reduce stress, alleviating excessive water intake. Thirdly, the supplemented mice did not show a significant decrease in total distance observed in the unsupplemented sCSDS mice (Table 1). Total ambulatory distance is one of the most important markers for the exploratory behavior and spontaneous motor ability of mice in an open field test. Increased anxiety leads to less locomotion, thereby reducing the distance explored by mice. The SI test, but not the open field test, was conducted in this study; however, oral administration of TMC3115 may have alleviated the stress-induced anxiety-like emotion and suppressed the decrease in spontaneous locomotor activity. In addition, although there was no significant difference between the tested groups, the ratio of stress-resilient mice increased with TMC3115 supplementation (Table 1). In summary, these findings suggest that TMC3115 supplementation at least contributes to stress reduction.

Probiotics may affect the host’s neurological and psychiatric function by changing the gut microbiota [13]. In particular, it is reported that the emergence of *Bifidobacterium* in the gut may lead to the host’s stress resilience [16]. Therefore, we initially hypothesized that bifidobacteria supplementation would change gut microbiota and confer stress tolerance in the host mice. TMC3115 supplementation actually increased the genus *Bifidobacterium* in the feces and cecal contents. However, *Bifidobacterium* was also detected in the cecum of the unsupplemented groups. Thus, we analyzed the detected *Bifidobacterium* at the species level, although at low resolution. As a result, *Bifidobacterium pseudolongum* was mostly accounted for, and *B. bifidum* was only slightly detected in the mice supplemented with TMC3115 (Figure S1). Since *B. bifidum,* including TMC3115, show quite a low affinity to intestinal mucin from animals [24], they would not colonize in the murine gut. On the other hand, *B. pseudolongum* is widely distributed in animals, including rodents, as commensal bacteria [35]. Therefore, TMC3115 may confer stress resistance to the host by increasing the host’s indigenous bifidobacteria. In addition, bifidobacteria, especially from infants, mediate gut–brain communication by producing several metabolites (e.g., indole-3-lactate, a tryptophan-derived metabolite) that are important for neurodevelopment [36]. Although we could not measure such metabolites in this study, supplemented TMC3115 may be involved in stress resilience via its metabolites.

The most noteworthy finding of this study is that TMC3115 supplementation remarkably suppressed the stress-induced IL-6 production (Figure 5). It is reported that stressful experiences and negative emotions can directly stimulate the production of IL-6 and other proinflammatory cytokines [37,38]. The increased production of proinflammatory cytokines has been proposed as one of the mechanisms for the development of depression [39], which is referred to as the inflammatory hypothesis [40]. Several studies that report the correlation between depressed patients and proinflammatory markers such as IL-6, IL-1βα, TNF-α, and C-reactive protein (CRP) so far support this hypothesis [41,42]. A cumulative meta-analysis also indicates a significant association between major depression and the level of IL-6 [43]. Therefore, IL-6 is identified as a novel target for major depressive disorder treatment [44]. Considering that stress and IL-6 are closely related to the development of depressive orders, TMC3115 may be a new candidate substance for the prevention or treatment of depression in the future.

Stress may be associated not only with inflammation, as mentioned above, but also with the abundance of *Proteobacteria*. Langgartner et al., previously reported an expansion of *Proteobacteria*, especially *Helicobacter* spp., in another rodent model for chronic psychosocial stress [45]. *Proteobacteria* often increase in various diseases, mostly with an inflammatory phenotype [46]. In fact, we observed that the increased *Proteobacteria* mostly accounted for the unclassified genus belonging to the *Desulfovibrionaceae* family ([f]_*Desulfovibrionaceae*) and that proinflammatory cytokines in the mice were subjected to sCSDS (Figure 3 and Figure 5). Thus, we analyzed the correlation between the serum proinflammatory cytokine levels and cecal microbiota (Table S3). As a result, the serum TNF-α level positively correlated with the phylum *Proteobacteria* and the genera [f]_*Desulfovibrionaceae* and *Desulfovibrio*. In contrast, there was no correlation between the serum IL-6 level and these genera, as estimated from the fact that TMC3115 supplementation did not affect the abundance of *Proteobacteria*. Additionally, no correlation between the serum IL-6 level and even the genus *Bifidobacterium* was observed. These findings indicate that TMC3115 modulates IL-6 production without involving gut microbiota. Suzuki et al., reported that TMC3115 might suppress IL-6 production from preadipocytes via stimulating mononuclear cells from Peyer’s patch using a clonal porcine intramuscular preadipocyte line [47]. Therefore, the supplemented TMC3115 may have directly modulated the host’s intestinal immune system and suppressed IL-6 production.

In conclusion, oral supplementation of probiotic TMC3115 was associated with stress tolerance in the host. The possible mechanisms were the increase in bifidobacteria and the anti-inflammation associated with a low peripheral IL-6 level. In addition, the effect of reducing the peripheral IL-6 level shows potential for the prevention and treatment of depression in the future. Further studies are required to confirm these findings.

## Figures and Tables

**Table 1:** Summary of SI test.

		Ct	St	TMC3115	*p*-Value
Susceptible (SI score <100)	No. (%)	3 (15.0%)	8 (33.3%)	5 (20.0%)	0.131 ^β^
Resilient (SI score ≥100)	No (%)	17 (85.0%)	16 (66.7%)	20 (80.0%)	
Total distance (cm) ^α^	ICR(−)	1076.0 ± 78.6	901.6 ± 39.5	948.0 ± 40.9	Ct vs. St: 0.066 ^γ^
	ICR(+)	642.0 ± 32.1	491.8 ± 34.4	544.2 ± 33.3	Ct vs. St: 0.008 ^γ^
Interaction zone time (s) ^α^	ICR(−)	46.9 ± 4.7	43.6 ± 3.5	42.2 ± 2.6	0.643 ^δ^
	ICR(+)	67.2 ± 5.3	57.2 ± 5.1	55.0 ± 4.1	0.524 ^δ^
Corner zone time (s) ^α^	ICR(−)	28.4 ± 4.2	28.7 ± 3.1	33.1 ± 2.9	0.191 ^δ^
	ICR(+)	26.8 ± 4.7	30.6 ± 5.7	36.1 ± 5.2	0.463 ^δ^

^α^ Data are expressed as mean ± SEM. ^β^ Chi-square test was performed on a 2 × 3 cross table. ^γ^ *p* value analyzed using Tukey’s post hoc test. ^δ^ *p* value analyzed using one-way ANOVA.

## Data Availability

The data presented in this study are available from the corresponding author upon request.

## Supplementary Materials

The following supporting information can be downloaded at: https://www.mdpi.com/article/10.3390/nu14050970/s1, Figure S1: Analysis of bifidobacteria at the species level; Table S1: Composition of FG medium; Table S2: Ingredients and nutrients of AIN-93G; Table S3: Correlation between the inflammatory cytokines and cecum microbiota.
