# Supplementary material for: Effect of Probiotic Bifidobacterium bifidum TMC3115 Supplementation on Psychosocial Stress Using a Sub-Chronic and Mild Social Defeat Stress in Mice
Source: Nutrients. 2022 Feb 24;14(5):970. doi: 10.3390/nu14050970 (PMC8912754; doi:10.3390/nu14050970)
Supplement: Supplementary file 1 [file nutrients-14-00970-s001.zip › nutrients-1577944-supplementary.pdf]

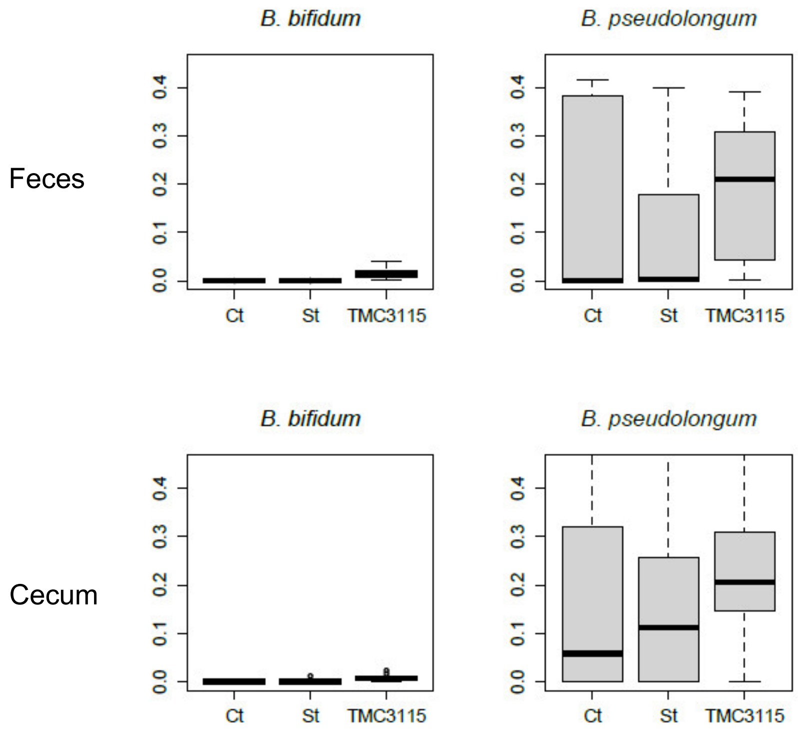

Figure S1. Analysis of bifidobacteria at the species level.

Table S1. Composition of FG medium.

Ingredients	g/L
Glucose	20.0
Peptone	20.0
Yeast Extract	5.0
Sodium acetate	5.0
Diammonium hydrogenphosphate	2.0
Dipotassium Hydrogenphosphate	2.0
Magnesium sulfate	0.5
Polysorbate 80	0.1

Table S2. Ingredients and nutrients of AIN-93G.

Ingredients (%)		Nutrients (%)	
Casein	20.0	Protein	17.9
L-Cystein	0.3	Fat	7.0
Corn starch	52.9	Ash	2.5
Sucrose	10.0	Fiber	3.0
Soy oil	7.0	Nitrogen free extract	60.6
Cellulose powder	5.0		
AIN-93 mineral mix	3.5		
AIN-93 vitamin mix	1.0		
Choline bitartrate	0.25		
Thertiary butyl hydroquinone	0.001		
Total	100.0	Calorie (kcal/100 g)	377.0

Data from Oriental Yeast, Tokyo, Japan.

Table S3. Correlation between the inflammatory cytokines and cecum microbiota.

	correlation coefficient (r_S)	
	IL-6*	TNF-α
<Phylum>		
Actinobacteria	-0.048 (0.041)	0.082
Bacteroidetes	-0.037 (0.095)	-0.134
Deferribacteres	-0.114 (0.096)	0.209
Firmicutes	-0.091 (-0.275)	-0.237
Proteobacteria	0.197 (0.453)	0.492
<Genus>		
Bifidobacterium	-0.042 (0.047)	0.081
Adlercreutzia	-0.047 (0.031)	0.097
Bacteroides	-0.132 (0.012)	-0.303
Mucispirillum	-0.114 (0.096)	0.209
Bilophila	0.039 (0.132)	0.228
[f]_Desulfovibrionaceae	0.193 (0.354)	0.499
Desulfovibrio	0.184 (0.505)	0.530

Analyzed using Spearman's rank correlation.

*The numbers in parentheses are the correlation coefficients when the TMC3115 group is excluded.
